# Synergistic Bactericidal Efficiency of Slightly Acidic Electrolyzed Water–High-Pressure Parallel Processing on *Escherichia coli* in Freshly Cut *Gastrodia elata* Slices

**DOI:** 10.3390/foods14050790

**Published:** 2025-02-25

**Authors:** Qing Gao, Xin Nong, Tuanjian Lang, Yajin Liu, Shuxin Ye, Jinsong He

**Affiliations:** 1 College of Food Science and Technology, Yunnan Agricultural University, Kunming 650201, China; 2015053@ynau.edu.cn (Q.G.); nx18008765177@163.com (X.N.); langtuanjian888@163.com (T.L.); 2 Kunming Tian Tian Xiang Shang Central Kitchen Operation Management Co., Ltd., Kunming 650220, China; 13388858495@163.com

**Keywords:** acidic electrolyzed water (SAEW), high-pressure (HP), green bactericidal technique, sterilization process optimization, microorganism control

## Abstract

The synergistic enhancement of bactericidal efficiencies on freshly cut *Gastrodia elata* slices by parallel processing using slightly acidic electrolyzed water (SAEW) and high-pressure (HP) technology was comprehensively investigated in this study. To this end, appropriate experimental conditions were determined through single-factor tests, which were ACCs (available chlorine concentrations) of 30, 38, and 49 mg/L; pressures of 100, 150, and 200 MPa; treatment times of 5, 7.5, and 10 min; and material-to-liquid ratios of 1:1, 1:3, and 1:5. Under these conditions, single and parallel bactericidal tests were conducted, and the corresponding synergistic enhancement values Δ*I* were calculated. Subsequently, using the lethal rate of *Escherichia coli* (*E. coli*) as the response value, we fitted multiple quadratic regression equations for SAEW, HP, and SAEW–HP with respect to ACC, pressure, pressure application time, and material-to-liquid ratio. The multiple quadratic regression equation for the synergistic enhancement term Δ*I* was then obtained through calculation. By analyzing this equation, the synergistic enhancement range was determined. Finally, experimental points were randomly selected within the synergistic enhancement range for validation. The results demonstrate that there was a synergistic bactericidal efficiency of the SAEW–HP parallel treatment of freshly cut *G. elata* slices. The synergistic enhancement range was pressure (x*_p_*) ∈ [52.18, 359.58] MPa; concentration of available chlorine (x*_c_*) ∈ [28.71, 46.27] mg/L; time (x*_t_*) ∈ [2.34, 12.38] min; and the material-to-solvent ratio (x*_r_*) ∈ ø g/mL. The validation experiments confirmed that within the respective ranges of *p*, *c*, and *t*, the SAEW–HP parallel treatment of freshly cut *G. elata* slices exhibited a ‘1 + 1 > 2’ synergistic enhancement effect. These findings lay a theoretical foundation for the development of green bactericidal technologies for “adopting both minimum processing and dosage to achieve the optimal effect”.

## 1. Introduction

Fresh vegetables and fruits are associated with the occurrence of various foodborne diseases. The microbial contamination level of freshly cut ready-to-eat fruits and vegetables is much higher than that of unprocessed ones. The browning of freshly cut fruits and vegetables is mostly caused by enzymatic browning. The main pathogenic microorganisms in freshly cut fruits and vegetables are *Staphylococcus aureus*, *Escherichia coli* (*E. coli*), *molds*, etc. [1,2,3]. *Gastrodia elata* (*G. elata*), a perennial herb of the Orchidaceae family, was included in November 2023 in the directory of substances for both food and traditional Chinese medicinal materials [4]. Consequently, the demand for *G. elata* as a fresh food product has significantly increased. However, fresh *G. elata* products are prone to microbial contamination during storage and transportation, which influences their quality [5]. Therefore, the issue of microbial control during the storage and transportation of fresh *G. elata* products needs to be addressed urgently.

Traditional processing methods for *G. elata* include cooking, honey processing, wine processing, drying, thermal processing, as well as non-thermal processing and bactericidal techniques. Each of these techniques has varying impacts on the quality of *G. elata* [6]. Thermal processing techniques can lead to the loss of sensory characteristics and nutrients of food products during heating [7,8]. Common non-thermal processing techniques include those using acidic electrolyzed water, high-pressure, pulsed electric fields, and high-pressure carbon dioxide. Among them, treatment methods such as those using pulsed electric fields and high-pressure carbon dioxide are still unable to completely eliminate microorganisms [9,10]. Slightly acidic electrolyzed water (SAEW) shows excellent bactericidal effects and is colorless, odorless, and highly safe [11,12,13]. Numerous studies have shown that SAEW has a relatively good bactericidal efficiency on freshly cut cabbage, lettuce, red pears, ham, rice noodles, freshly cut apples, freshly cut eggplants, carp, beef, and wild mushrooms [14,15,16,17,18,19,20,21,22,23,24]. However, SAEW is unstable and easily decomposes, thus limiting its solo application to some extent. The high-pressure (HP) technique is commonly used for bactericidal treatment, processing, and enzyme inactivation in food products. It can improve the taste and flavor of freshly cut fruits and vegetables, maintain relatively good sensory quality during long-term storage, enhance the inherent defense capabilities of fruits and vegetables, reduce the incidence of diseases during storage, and improve the storage quality of freshly cut fruits and vegetables [8,25,26,27,28]. However, the HP technique is costly and has limited application scenarios.

The bactericidal effect of a single technique is limited despite the fact that it exerts certain benefits for the preservation and freshness of food products. Existing studies have indicated that combining single techniques in parallel produce a synergistic effect (parallel processing effect is superior to single techniques) [16,29,30,31]. However, the correlation and comparison between the effects of single and parallel techniques have not been quantitatively elucidated. In this study, the objective was to investigate the bactericidal effect of SAEW-HP on freshly cut *Gastrodia elata* slices. *Escherichia coli* was used as a model bacterium and the bactericidal rate as a response indicator to explore whether the SAEW–HP parallel bactericidal technique exhibits a ‘1 + 1 > 2’ synergistic effect. The findings aim to lay a theoretical foundation for the development of novel bactericidal techniques that meet the green processing concept of “adopting both minimum processing and dosage to achieve the optimal effect”. Furthermore, the related results can provide a theoretical basis for the production and processing of freshly cut *G. elata* slices.

## 2. Materials and Methods

### 2.1. Experimental Raw Materials

We activated the freeze-dried powder of *E. coli* ATCC 25922, incubated it in a 37 °C shaker (63R, Shanghai Yanquan Co., Ltd., Shanghai, China) for 24 h, and stored it in a refrigerator (BCD-4482P9CX, Hefei Meiling Stock Company, Hefei, China) at 4 °C. The strain was transferred to a new culture medium every week to maintain its activity. Each time the bacterial strain was used, a ring of bacterial solution was taken from the inoculation ring and inoculated into a new broth culture. The mixture was then placed in a constant-temperature shaker and cultured at 37 °C for 24 h. This process was repeated three times in a row to achieve a viable bacterial count of 10^9^ CFU/mL.

### 2.2. Preparation and Inoculation Method of Freshly Cut G. elata Slices

Fresh *G. elata* without diseases or pests was selected, washed with tap water, dried, peeled, sliced, and then stored at room temperature (25 °C). The slices were 5 mm in thickness and were pressed into 2 cm diameter disks using a cylindrical mold, and 5.0 ± 0.5 g of the freshly cut slices was placed in Petri dishes (Φ 90 * 90 mm). They were then subjected to UV irradiation for 15 min on each side for the bactericidal treatment.

Referencing the methods of M. Abadias [32] and A. Issazacharia et al. [33] with slight modifications, we immersed the freshly cut *G. elata* slices in 10 mL of bacterial suspension with a concentration of approximately 10^7^ CFU/mL at room temperature (25 °C) for 10 min while stirring continuously.

After removal, the slices were allowed to drain for 30 min on a clean bench (SW-CJ-2FD, Suzhou Antai Air Technology Co., Ltd., Suzhou, China). The surface bacterial count of the freshly cut *G. elata* slices was approximately 10^5^ CFU/g.

### 2.3. Preparation of SAEW

A 6% HCl solution was prepared at room temperature (25 °C) and processed through an SAEW generator (HD-240L, Want Want Group, Shanghai, China) to produce SAEW. The prepared SAEW was collected at room temperature (25 °C) in a lightproof container for experimental use on the same day. By changing the current (*I*), SAEW with different available chlorine concentrations (ACCs) could be prepared. The determination of ACC was performed using the iodometric method [34]. A standard curve was plotted with *I* as the abscissa and ACC as the ordinate. The standard curve equation was *R*^2^ = 0.99.

### 2.4. Single-Factor Experiment

#### 2.4.1. SAEW Bactericidal Treatment

##### Determination of ACC of SAEW

With a fixed material-to-liquid ratio of 1:3 g/mL, a pressure of 0.1 MPa, a treatment time of 10 min, and a room temperature of 25 °C, the ACC of SAEW was varied (24 mg/L, 30, 38, and 49 mg/L), and the total number of colonies was measured after treatment.

##### Determination of Treatment Time of SAEW

With a fixed ACC of SAEW of 49 mg/L, a pressure of 0.1 MPa, a material-to-liquid ratio of 1:3 g/mL, and a room temperature of 25 °C, the treatment time of SAEW was varied (0, 2.5, 5, 7.5, and 10 min), and the total number of colonies was measured after treatment.

##### Determination of Material-to-Liquid Ratio of SAEW

With a fixed ACC of SAEW of 49 mg/L, a pressure of 0.1 MPa, a treatment time of 10 min, and a room temperature of 25 °C, the SAEW material-to-liquid ratio was varied (1:1, 1:3, and 1:5 g/mL), and the total number of colonies was measured after treatment.

#### 2.4.2. HP Bactericidal Treatment

##### Determination of HP

With a fixed material-to-liquid ratio of 1:3 g/mL, an ACC of SAEW of 0 mg/L, a treatment time of 10 min, and a room temperature of 25 °C, the pressure was varied (0.1, 50, 100, 150, and 200 MPa) HP (HPP.L1-600/5, Tianjin Huatengmiao Biological Engineering Technology Co., Ltd., Tianjin, China), and the total number of colonies was measured after treatment.

##### Determination of HP Treatment Time

With a fixed material-to-liquid ratio of 1:3 g/mL, an ACC of SAEW of 0 mg/L, a pressure of 300 MPa, and a room temperature of 25 °C, the treatment time was varied (0, 2.5, 5, 7.5, and 10 min), and the total number of colonies was measured after treatment.

##### Determination of HP Material-to-Liquid Ratio

With a fixed treatment time of 10 min, an ACC of SAEW of 0 mg/L, a pressure of 300 MPa, and a room temperature of 25 °C, the material-to-liquid ratio was varied (1:1, 1:3, and 1:5 g/mL), and the total number of colonies was measured after treatment.

#### 2.4.3. Response Surface Design

Based on the single-factor experiments, four factors were selected for the response surface test: material-to-liquid ratio, ACC, treatment time, and pressure, with the number of dead colonies as the response value. The optimal combination of the four factors was used to explore the bactericidal efficiency of SAEW combined with high pressure on freshly cut *G. elata* slices. The levels and codes of the SAEW, HP, and SAEW-HP test factors are detailed in Table 1, Table 2 and Table 3.

#### 2.4.4. Measurement of Total Number of Colonies

The total number of colonies was measured in line with the national standard GB 4789.2-2022, ‘Food Microbiological Inspection Determination of Total Bacterial Colony’ [35]. A 5.0 ± 0.5 g sample of freshly cut *G. elata* slices was placed in 45 mL of physiological saline after bactericidal treatment and shaken thoroughly. Different dilution gradients were selected, and 1 mL of eluent was added. Subsequently, 15–20 mL of plate counting medium was poured, and incubation was carried out for 48 h (DHP-600, Beijing Yongguang Medical Instrument Co., Ltd., Beijing, China) for reading.

### 2.5. Construction Method for the Bactericidal Synergy Term Model

By constructing a multivariate quadratic equation for the synergy terms related to material-to-liquid ratio, SAEW, HP, and time, the response surface method was used to identify the enhancement area and elucidate the synergistic effects during the SAEW and HP parallel bactericidal treatment process. In accordance with Equation (1), we placed freshly cut *G. elata* slices inoculated with *E. coli* after bactericidal treatment under atmospheric pressure in SAEW with different material-to-liquid ratios and ACCs for varying durations. The experimental data were fitted using multiple regression to obtain a multivariate quadratic regression equation for the bactericidal efficiency of *E. coli* (*Y*_SAEW_) in terms of the variables ACC, soaking time, and material-to-liquid ratio. The freshly cut *G. elata* slices inoculated with *E. coli* after bactericidal treatment were then subjected to different pressures for varying times in sterile water with different material-to-liquid ratios, and the experimental data were fitted with multiple regression to obtain a multivariate quadratic regression equation for the bactericidal efficiency of *E. coli* (*Y*_HP_) in terms of the variables’ pressure, pressurization time, and material-to-liquid ratio. The freshly cut *G. elata* slices inoculated with *E. coli* were treated using the parallel technique (i.e., soaking the freshly cut *G. elata* slices in SAEW with different material-to-liquid ratios and ACCs under different pressures for varying times), and the experimental data were fitted with multiple regression to obtain a multivariate quadratic regression equation for the bactericidal efficiency of *E. coli* (*Y*_SAEW-HP_) in terms of the variables ACC, soaking time, material-to-liquid ratio, and pressure. According to Equation (4), the obtained bactericidal efficiencies *Y*_SAEW-HP_, *Y*_HP_, and *Y*_SAEW_ were substituted into Equation (1) to derive the multivariate quadratic equation for the synergy term Δ*I* in terms of the variables ACC, soaking time, material-to-liquid ratio, and pressure.

The general form of the multivariate quadratic equation is as follows:

(1) $$Y=\alpha +\sum \beta_{i}x_{i}+\sum \gamma_{jk}x_{j}x_{k}$$

where *α* is a constant term, *β_i_* is a constant term with respect to *x_i_*, *γ_jk_* is a constant term with respect to *x_i_ x_k_*, *i* ∈ {concentration of available chlorine (*c*); pressure (*p*); time (*t*); material-to-liquid ratio (*r*)}, *j* ∈ {concentration of available chlorine (*c*); pressure (*p*); time (*t*); material-to-liquid ratio (*r*)}, and *k* ∈ {concentration of available chlorine (*c*); pressure (*p*); time (*t*); material-to-liquid ratio (*r*)}.

Under the condition of the same initial microbial content, the bactericidal efficiency is represented by *I*. For the calculation, the following formula is used:

(2) I=−ln⁡N

where *I* is the bactericidal efficiency after treatment under the treatment conditions, and *N* is the total number of colonies (CFU/g) after treatment under the treatment conditions.

(3) $$∆I=I_{SAEW-HP}-(I_{SAEW}+I_{HP})$$

where Δ*I* is the synergy term, *I*_SAEW_ is the lethal rate after SAEW treatment, *I_HP_* is the lethal rate after HP treatment, and *I*_SAEW-HP_ is the lethal rate after parallel treatment.

By combining Equations (2) and (3), the calculation formula for the synergy term Δ*I* can be derived as follows:

(4) $$∆I=-\ln N_{SAEW-HP}+\ln N_{SAEW}+\ln N_{HP}$$

where Δ*I* is the synergy term, *N*_SAEW-HP_ is the total number of colonies (CFU/g) after parallel treatment, *N*_SAEW_ is the total number of colonies (CFU/g) after SAEW treatment, and *N*_HP_ is the total number of colonies (CFU/g) after HP treatment.

When Δ*I* > 0, the bactericidal efficiency of the parallel action of the two techniques is superior to the sum of their bactericidal efficiencies during individual treatment, meaning that the SAEW and HP bactericidal treatments have an enhanced effect of ‘1 + 1 > 2’. When Δ*I* = 0, meaning ‘1 + 1 = 2’, this indicates that the parallel treatment effect is equal to the sum of the bactericidal efficiencies of SAEW and HP during individual treatments. When Δ*I* < 0, the bactericidal efficiency of the parallel action of the two techniques is lower than the sum of bactericidal efficiencies during individual treatments; thus, 1 + 1 < 2, indicating that the parallel bactericidal efficiency does not surpass that of a single technique, or that the parallel bactericidal efficiency is stronger than that of a single technique but less than the sum of the effects of the two techniques during individual treatment, thus failing to achieve an enhanced effect and showing no synergistic effect.

### 2.6. Data Analysis

Graphing and nonlinear fitting analysis were conducted using Origin software (2021), response surface analysis was performed using Design-Expert (13), and significance analysis of the data was carried out using SPSS software (26).

## 3. Results and Discussion

### 3.1. The Effect of Different Treatment Methods on the Bactericidal Efficiency Against E. coli on the Surfaces of Freshly Cut G. elata Slices

**Figure 1:**
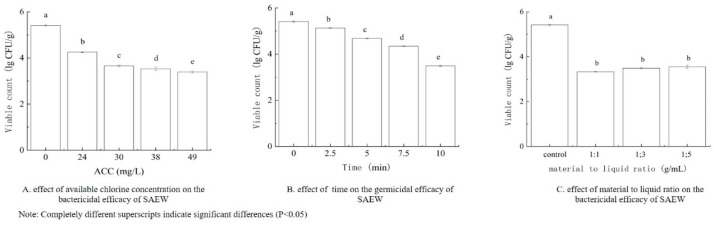
Effect of various factors on the bactericidal efficiency of SAEW. (**A**) Effect of available chlorine concentration on the bactericidal efficiency of SAEW, (**B**) Effect of time on the germicidal efficiency of SAEW, (**C**) Effect of material to liquid ratio on the bactericidal efficiency of SAEW. Note: Completely different superscripts indicate significant differences (*p* < 0.05).

**Figure 2:**
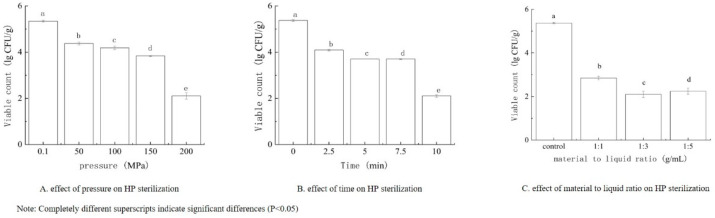
Effect of various factors on the bactericidal efficiency of HP. (**A**) Effect of pressure on HP sterilization, (**B**) Effect of time on HP sterilization, (**C**) Effect of material to liquid ration on HP sterilization. Note: Completely different superscripts indicate significant differences (*p* < 0.05).

Based on the single-factor tests shown in Figure 1, ACCs of 30, 38, and 49 mg/L were selected, along with treatment times of 5, 7.5, and 10 min and material-to-liquid ratios of 1:1, 1:3, and 1:5 g/mL as experimental points for the subsequent tests. From the single-factor tests shown in Figure 2, pressures of 100, 150, and 200 MPa were chosen, along with treatment times of 5, 7.5, and 10 min, and material-to-liquid ratios of 1:1, 1:3, and 1:5 g/mL as experimental points for the subsequent tests.

**Figure 3:**
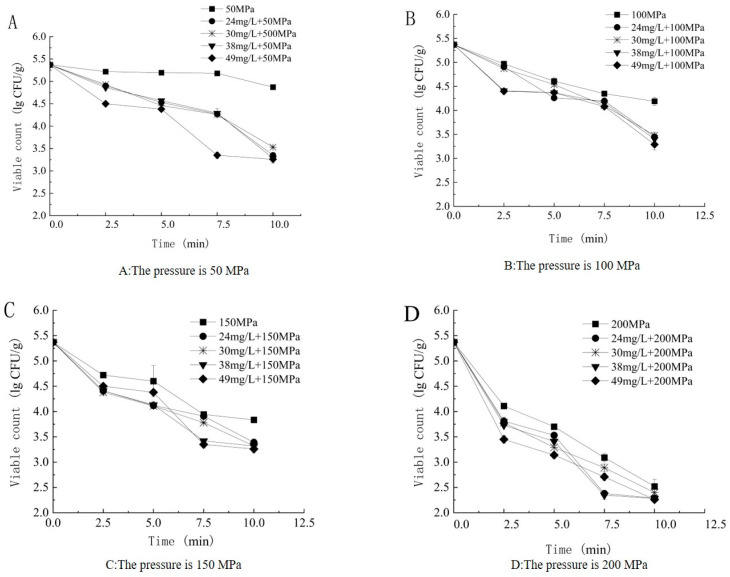
Different ACCs changed under the same HP conditions. (**A**) The pressure is 50 MPa, (**B**) The pressure is 100 MPa, (**C**) The pressure is 150 MPa, (**D**) The pressure is 200 MPa.

As indicated in Figure 3, under fixed pressure and varying ACC conditions, the bactericidal efficiency increased with the increase in ACC. Moreover, the synergistic enhancement term ∆*I* was calculated according to Equations (2) and (3), with ∆*I* > 0. This suggests that the SAEW–HP parallel bactericidal efficiency was superior to the bactericidal efficiency of an individual technical treatment, indicating the presence of a synergistic enhancement effect.

**Figure 4:**
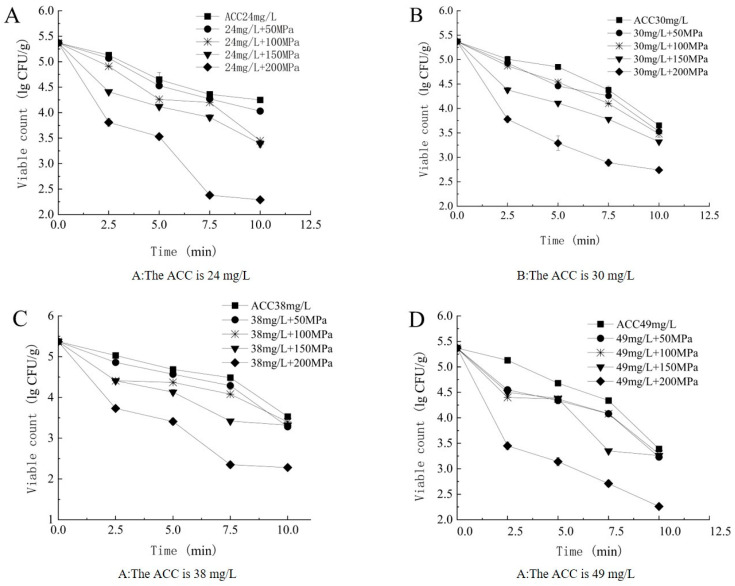
Different HP changed under the same ACC conditions. (**A**) The ACC is 24 mg/L, (**B**) The ACC is 30 mg/L, (**C**) The ACC is 38 mg/L, (**D**) The ACC is 49 mg/L.

As shown in Figure 4, under fixed ACCs and varying pressure conditions, the bactericidal efficiency increased with the increase in pressure. The synergistic enhancement term ∆*I* was also calculated according to Equations (2) and (3), with ∆*I* > 0. This indicates that the SAEW–HP parallel bactericidal efficiency was superior to the bactericidal efficiency of the individual technical treatment, confirming the presence of a synergistic enhancement effect.

In summary, after tests at the relevant experimental points identified in the single-factor tests, it was found that the SAEW–HP parallel treatment had a synergistic enhancement of bactericidal efficiency against *E. coli*.

### 3.2. Establishment of the Synergistic Bactericidal Prediction Model

#### 3.2.1. Analysis of the Response Surface Results for the Bactericidal Efficiency of SAEW on Freshly Cut *G. elata* Slices

Based on the single-factor experiments, a response surface test was conducted using the Box–Behnken design principle, with ACC (*c*), soaking time (*t*), and material-to-liquid ratio (*r*) as independent variables, and the viable count of freshly cut *G. elata* slices (*Y*_SAEW_) as the response value. The data were analyzed, and the regression equation for the bactericidal efficiency of SAEW on freshly cut *G. elata* slices was derived according to Equation (1). The pressure (*p*) was kept at 0.1 MPa, with *Xp* = 0, as follows:

$$Y_{SAEW}=7.49+0.044x_{c}+0.88x_{t}+0.59x_{r}+0.0014x_{c}x_{t}+0.0053x_{c}x_{r}-0.016x_{t}x_{r}-0.00094x_{c}^{2}-0.097x_{t}^{2}-0.12x_{r}^{2}$$

The model was subjected to an analysis of variance, and the obtained multiple regression model was found to be highly significant (*p* < 0.01). Furthermore, *R*^2^ was found to be 0.94, which indicates that the regression equation had a good fit. The response surface fitting formula could reflect the experimental results quite accurately and was applicable for predicting the bactericidal efficiency of SAEW on freshly cut *G. elata* slices.

#### 3.2.2. Analysis of the Response Surface Results for the Bactericidal Efficiency of HP on Freshly Cut *G. elata* Slices

Building on the single-factor experiments, we conducted a response surface test using the Box–Behnken design principle, with material-to-liquid ratio (*r*), treatment time (*t*), and pressure (*p*) as independent variables, and the viable count on freshly cut *G. elata* slices (*Y*_HP_) as the response value. The data were analyzed, and the regression equation for the bactericidal efficiency of the HP treatment on freshly cut *G. elata* slices was derived according to Equation (1). ACC(c) is 0 mg/L, with *Xc* = 0, as follows:

$$Y_{HP}=9.80+0.053x_{p}-0.091x_{t}-0.53x_{r}-0.0035x_{p}x_{t}+0.00058x_{p}x_{r}+0.048x_{t}x_{r}-0.00019x_{p}^{2}+0.006x_{t}^{2}+0.055x_{r}^{2}$$

The model was subjected to an analysis of variance, which revealed that the obtained multiple regression model was extremely significant (*p* < 0.01). *R*^2^ was 0.99, indicating that the regression equation had a good fit. The response surface could accurately reflect the experimental results and was applicable for predicting the bactericidal efficiency of HP on freshly cut *G. elata* slices.

#### 3.2.3. Response Surface Analysis of the Bactericidal Efficiency of SAEW–HP Parallel Treatment on Freshly Cut *G. elata* Slices

Using the Box–Behnken design principle, we conducted a response surface test with material-to-liquid ratio (*r*), treatment time (*t*), ACC of SAEW (*c*), and pressure (*p*) as independent variables, and the viable count (*Y*_SAEW-HP_) as the response value. The data were analyzed, and the regression equation for the bactericidal efficiency of the SAEW–HP parallel treatment on freshly cut *G. elata* slices was derived according to Equation (1) as follows:

$$Y_{SAEW-HP}=16.63-0.11x_{c}+0.037x_{p}-1.33x_{t}+0.043x_{r}-0.000068x_{c}x_{p}-0.0031x_{c}x_{t}+0.00026x_{c}x_{r}-0.0039x_{p}x_{t}-0.00055x_{p}x_{r}+0.056x_{t}x_{r}+0.0017x_{c}^{2}-0.00012x_{p}^{2}+0.099x_{t}^{2}-0.0978x_{r}^{2}$$

The model was subjected to an analysis of variance, which indicated that the obtained multiple regression model was extremely significant (*p* < 0.01). *R*^2^ was found to be 0.97, which indicates that the regression equation had a good fit. The response surface could accurately reflect the experimental results and was applicable for predicting the bactericidal efficiency of the SAEW–HP parallel treatment on freshly cut *G. elata* slices.

#### 3.2.4. Analysis of the Synergistic Enhancement Effect of SAEW–HP Parallel Bactericidal Treatment

Based on the response surface experiment of the parallel bactericidal treatment, we adopted the factors of ACC (*c*), pressure (*p*), time (*t*), and material-to-liquid ratio (*r*) for parallel bactericidal treatment. According to Equations (1) and (4), with Δ*I* as the response value, the regression equation was obtained through software analysis:

$$\Delta I=-3.19+0.18x_{c}+0.07x_{p}+1.62x_{t}+0.80x_{r}+0.000068x_{c}x_{p}-0.000737x_{c}x_{t}-0.0014x_{c}x_{r}-0.0027x_{p}x_{t}+0.000025x_{p}x_{r}-0.025x_{t}x_{r}-0.0024x_{c}^{2}-0.00017x_{p}^{2}-0.11x_{t}^{2}-0.089x_{r}^{2}$$

**Figure 5:**
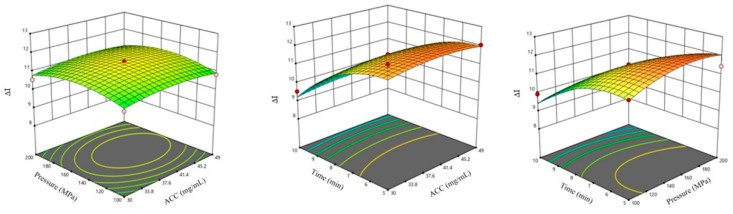
Response surface plot of the interaction effects of various factors on Δ*I* in SAEW–HP parallel treatment.

According to the analysis in Figure 5, the response surface plot of the interaction between pressure and ACC on Δ*I* is close to an ellipse, indicating that there is an interaction between the two. When the pressure is fixed, as ACC increases, Δ*I* gradually increases. When ACC is fixed, as the pressure increases, Δ*I* gradually increases. The maximum value of the model is *x_p_* = 169.48 MPa, with *x_c_* = 30.00 mg/L, *x_r_* = 4.03 g/mL, *x_t_* = 5.00 min, and Δ*I* =11.97 > 0.

Analysis of variance of the model revealed that the regression model was significant, with *p* < 0.05. The *R*^2^ of the model was 0.8484, indicating that the model had a good fit.

By using the multivariate quadratic equation of Δ*I*, the synergistic enhancement interval of ‘1 + 1 > 2’ for each influencing factor when Δ*I* > 0 could be determined. The calculated bactericidal enhancement intervals for the SAEW–HP parallel treatment of freshly cut *G. elata* slices were as follows: *p* ∈ [52.18, 359.58] MPa; *c* ∈ [28.71, 46.27] mg/L; *t* ∈ [2.34, 12.38] min; *r* ∈ ø g/mL. This indicates that within the corresponding interval ranges of *p*, *c*, and *t*, the parallel SAEW–HP treatment of freshly cut *G. elata* slices exhibited a “1 + 1 > 2” synergistic enhancement effect, while the material-to-liquid ratio *r* had no significant impact on the synergistic enhancement effect.

Current studies have shown that parallel bactericidal techniques, compared to a single bactericidal technique, have a good inhibitory effect on *Bacillus subtilis* in wolfberry juice, *E. coli* in strawberry juice, and spores in beef broth [29,30,36]. This is consistent with the findings of our study, which demonstrate that parallel treatment has a synergistic enhancement effect on microbial control, and that parallel bactericidal techniques can produce better bactericidal efficiencies than single bactericidal techniques. However, the existing literature only states that parallel bactericidal treatment is more effective than single bactericidal treatment without quantitatively characterizing the dose–efficiency relationship between parallel and single bactericidal techniques. Using the response surface method, this study accurately determines through the calculation and analysis of Δ*I* that there are certain intervals where the parallel bactericidal efficiency is superior to the sum of the efficiencies of single bactericidal techniques, i.e., there is a “1 + 1 > 2” synergistic enhancement effect.

Some scholars believe that green processing technology for food is based on traditional processing technology combined with various advanced mechanical control technology, biological processing technology, material science technology, and other high technology. It includes the reasonable use of resources, reduction in production costs, reduction in pollution and damage to the environment caused by processing, as well as the use of technology for the production of safe, healthy, nutritious, delicious, and not over-processed food [37,38,39]. This study used non-thermal processing technology instead of the hot processing method to treat *Gastrodia* fresh slices. This reduced the heat damage made to the quality of *Gastrodia* fresh slices and better retained the nutritional quality of *Gastrodia* fresh slices. In addition, through parallel processing with the two technologies, the dosages of SAEW and HP were reduced to achieve a better sterilization effect, and a new concept of green processing and green sterilization was realized.

In this study, the range of synergistic bactericidal efficiency of SAEW–HP parallel processing for *Escherichia coli* in freshly cut *Gastrodia elata* was found, and a method for it was established. This method provides a theoretical basis for actual production and processing. In the future, parallel processing technology will be combined with actual production to develop related equipment and to reduce the operating cost of enterprises.

### 3.3. Validation Experiment for the Optimized Conditions of Freshly Cut G. elata Slices

Considering practical operational conditions, we randomly selected the following conditions based on the optimal conditions and collaborative enhancement interval range. We made a random selection of the following conditions for the bactericidal validation experiment on freshly cut *G. elata* slices. After treatment of the freshly cut *G. elata* slices under these conditions, the total number of colonies was detected, and the results are presented Table 4.

Based on the range of values and the extreme points of each influencing factor, five combinations were selected for the validation experiment. All combinations predicted values of Δ*I* > 0. By comparison of the predicted values with the actual verification values, the experimental results have been validated. They indicate that we have established the correct prediction method. The results of all the experiments indicated that the parallel SAEW–HP bactericidal treatment, within the range of influencing factors, resulted in a higher bactericidal number than the sum of the bactericidal numbers achieved by the single techniques using SAEW and HP, i.e., Δ*I* > 0. This proves that the parallel SAEW–HP bactericidal treatment is more effective than the sum of the bactericidal efficiencies of the bactericidal treatments of freshly cut *G. elata* slices with a single technique, demonstrating a ‘1 + 1 > 2’ synergistic enhancement effect. Parallel bactericidal treatment can more effectively inhibit the growth of surface microorganisms on freshly cut *G. elata* slices, achieving the green bactericidal concept of “adopting both minimum processing and dosage to achieve the optimal effect”.

## 4. Conclusions

This study demonstrates that the SAEW-HP parallel treatment of freshly cut *Gastrodia elata* slices has a synergistic enhancement effect on microbial control, and that parallel bactericidal techniques can produce better bactericidal efficiencies than single bactericidal techniques. Within the specific conditional region (*x_p_* ∈ [52.18, 359.58] MPa; *x_c_* ∈ [28.71, 46.27] mg/L; *x_t_* ∈ [2.34, 12.38] min; *x_r_* ∈ ø g/mL), the parallel SAEW–HP bactericidal treatment is more effective than the sum of the bactericidal efficiencies of the bactericidal treatments of freshly cut *G. elata* slices with a single technique, exhibiting a “1 + 1 > 2” synergistic enhancement effect.

In summary, by combining chemical bactericidal methods (SAEW) with physical bactericidal methods (HP) and by simultaneously applying different bactericidal mechanisms to microorganisms, the numbers of microbial deaths are increased, while it is ensured that SAEW and HP operate under relatively mild conditions for the bactericidal treatment. This novel approach meets the green processing concept of “adopting both minimum processing and dosage to achieve the optimal effect”. This study also provides a theoretical basis for the development of a green bactericidal technique for freshly cut food as well as guidance in actual production and processing.

## Figures and Tables

**Table 1:** SAEW test factor levels and coding.

Coding	ACC (mg/L)	Soaking Time (min)	Material-to-Liquid Ratio (g/mL)	Pressure (MPa)
−1	30	5	1:1	0.1
0	38	7.5	1:3	
1	49	10	1:5	

**Table 2:** HP test factor levels and coding.

Coding	Pressure (MPa)	Pressurization Time (min)	Material-to-Liquid Ratio (g/mL)	ACC (mg/L)
−1	100	5	1:1	0
0	150	7.5	1:3	
1	200	10	1:5	

**Table 3:** SAEW-HP test factor levels and coding.

Coding	ACC (mg/L)	Pressure (MPa)	Pressurization Time (min)	Material-to-Liquid Ratio (g/mL)
−1	30	100	5	1:1
0	38	150	7.5	1:3
1	49	200	10	1:5

**Table 4:** Validation results of freshly cut *G. elata* slices.

Experiment No.	Pressure (MPa)	ACC (mg/mL)	Treatment Time (min)	Material-to-Liquid Ratio (g/mL)	Δ*I*
1	200	39.5	7.5	1:3	12.03
2	150	39.5	7.5	1:5	10.05
3	200	30	5	1:3	10.67
4	150	39.5	5	1:5	12.38
5	200	30	5	1:5	10.72

## Data Availability

The original contributions presented in this study are included in the article. Further inquiries can be directed to the corresponding authors.
